# The rate of the founder Jewish mutations in *BRCA1* and *BRCA2* in prostate cancer patients in Israel

**DOI:** 10.1054/bjoc.2000.1249

**Published:** 2000-07-24

**Authors:** A Vazina, J Baniel, Y Yaacobi, A Shtriker, D Engelstein, I Leibovitz, M Zehavi, A A Sidi, Y Ramon, T Tischler, P M Livne, E Friedman

**Affiliations:** Institute of Urology Rabin Medical Center, (Belinson Campus), Petach Tikvah; Departments of Urology, the Chaim Sheba Medical Center, Tel-Hashomer; Edith Wolfson Medical Center; Department of Oncology, Susanne Levy Gertner Oncogenetics Unit, Chaim Sheba Medical Center, Tel-Hashomer, 62521, Israel

## Abstract

Inherited predisposition occurs in 5–10% of all prostate cancer (CaP) patients, but the genes involved in conferring genetic susceptibility remain largely unknown. Several lines of evidence indicate that germline mutations in *BRCA1* and *BRCA2* might be associated with an increased risk for CaP. Three mutations in these two genes (185delAG and 5382InsC (*BRCA1*) and 6174delT (*BRCA2*) occur in about 2.5% of the general Ashkenazi population, and the 185delAG *BRCA1* mutation, in up to 1% of non-Ashkenazi Jews. In order to assess the contribution of these germline mutations to prostate cancer in Jewish Israeli patients, we tested 174 unselected prostate cancer patients (95 of Ashkenazi origin) for these mutations by PCR amplification and modified restriction enzyme digests. Patient’s age range was 45–81 years (median 66), and in 24 (14.4%) the disease was diagnosed prior to 55 years of age. Nineteen (11%) and 12 (6.9%) patients had a first or second degree relative with CaP or breast cancer, respectively. Overall, five mutation carriers were detected: 2/152 (1.3%) 185delAG, 2/104 (2%) 5382InsC, and 1/158 (0.6%) 6174delT. In all carriers, the disease was diagnosed after the age of 55, and only one of them had a family history of breast and CaP. In addition, no allelic losses at the *BRCA1* locus were demonstrated in 17 patients with a family history of CaP, using seven microsatellite markers. We conclude that the rate of the predominant Jewish *BRCA1* and *BRCA2* mutations in CaP patients does not significantly differ from that of the general population, and that mutational inactivation of the *BRCA1* is rare in familial CaP. Thus, germline *BRCA1* and *BRCA2* mutations probably contribute little to CaP occurrence, to inherited predisposition, and to early onset disease in Jewish individuals. © 2000 Cancer Research Campaign


*British Journal of Cancer* (2000) **83**(4), 463–466
© 2000 Cancer Research Campaign
doi: 10.1054/ bjoc.2000.1249, available online at http://www.idealibrary.com on IDEAL®

### The rate of the founder Jewish mutations in *BRCA1* and *BRCA2* in prostate cancer patients in Israel

**A Vazina,[1] J Baniel,[1] Y Yaacobi,[3] A Shtriker,[3] D Engelstein,[1] I Leibovitz,[2] M Zehavi,[4] AA Sidi,[3] Y Ramon,[2] T Tischler,[4] PM Livne[1] and E Friedman[5]**

[1]Institute of Urology Rabin Medical Center (Belinson Campus), Petach Tikvah; [2]Departments of Urology, the Chaim Sheba Medical Center, Tel-Hashomer; [3]Edith Wolfson Medical Center; [4]Department of Oncology; [5]Susanne Levy Gertner Oncogenetics Unit, Chaim Sheba Medical Center, Tel-Hashomer, 62521, Israel

**Summary** Inherited predisposition occurs in 5–10% of all prostate cancer (CaP) patients, but the genes involved in conferring genetic susceptibility remain largely unknown. Several lines of evidence indicate that germline mutations in *BRCA1* and *BRCA2* might be associated with an increased risk for CaP. Three mutations in these two genes (185delAG and 5382InsC (*BRCA1*) and 6174delT (*BRCA2*) occur in about 2.5% of the general Ashkenazi population, and the 185delAG *BRCA1* mutation, in up to 1% of non-Ashkenazi Jews. In order to assess the contribution of these germline mutations to prostate cancer in Jewish Israeli patients, we tested 174 unselected prostate cancer patients (95 of Ashkenazi origin) for these mutations by PCR amplification and modified restriction enzyme digests. Patient's age range was 45–81 years (median 66), and in 24 (14.4%) the disease was diagnosed prior to 55 years of age. Nineteen (11%) and 12 (6.9%) patients had a first or second degree relative with CaP or breast cancer, respectively. Overall, five mutation carriers were detected: 2/152 (1.3%) 185delAG, 2/104 (2%) 5382InsC, and 1/158 (0.6%) 6174delT. In all carriers, the disease was diagnosed after the age of 55, and only one of them had a family history of breast and CaP. In addition, no allelic losses at the *BRCA1* locus were demonstrated in 17 patients with a family history of CaP, using seven microsatellite markers. We conclude that the rate of the predominant Jewish *BRCA1* and *BRCA2* mutations in CaP patients does not significantly differ from that of the general population, and that mutational inactivation of the *BRCA1* is rare in familial CaP. Thus, germline *BRCA1* and *BRCA2* mutations probably contribute little to CaP occurrence, to inherited predisposition, and to early onset disease in Jewish individuals. © 2000 Cancer Research Campaign

Prostate cancer (CaP) is a common cancer, and histopathological evidence of this cancer is found in up to 50% of men between 70–80 years of age (Sheldon et al, 1980), but in only a fraction of these, the disease becomes symptomatic and has clinical relevance (Gittes, 1991). Identifying individuals at risk for developing this common malignancy has obvious clinical applications, from both personal and national health care perspectives. Familial clustering and early onset disease are well known risk factors predisposing to CaP. The relative risk (RR) for developing CaP in first degree relatives of CaP patients ranges from 1.65 to 3.3, with increased RR associated with an earlier age at diagnosis and having more than one affected family member (Carter et al, 1992; Whittemore et al, 1995). These observations suggest an inherited predisposition to CaP, which seems to occur in about 5–10% of all CaP patients (Carter et al, 1992). The genes that underlie this genetic susceptibility remain largely unknown. Clustering of breast, ovarian and prostate cancer has been reported (Tulinius et al, 1992; Anderson and Badzioch, 1993; Sellers et al, 1994; Easton et al, 1997), suggesting a role for *BRCA1* (Arason et al, 1993; Briana et al, 1996) and *BRCA2* (Struewing et al, 1997) genes in CaP predisposition. Indeed, epidemiological studies estimated the RR for developing CaP in *BRCA1* mutation carriers is 3.33 (Ford et al, 1994, Easton et al, 1995), and that of first degree relatives of *BRCA2* mutation carriers at 4.6 (Sigurdsson et al, 1997). Among Jewish people, three predominant mutations within these two genes occur: 185delAG, 5382InsC (*BRCA1*) and 6174delT (*BRCA2*). These three mutations occur in the majority of individuals at risk for developing breast and ovarian cancer, and also in about 2.5% of the general Ashkenazi (East European) Jews, and the 185delAG mutation in up to 1% of non-Ashkenazi Jews (Bar-Sade et al, 1998). Indeed, the lifetime risk for developing CaP in Ashkenazi *BRCA1* or *BRCA2* mutation carriers was estimated at 16% (Struewing et al, 1997). However, direct mutational analysis of 60 unselected Jewish Ashkenazi CaP patients did not reveal any *BRCA1* or *BRCA2* mutation carrier (Lehrer et al, 1998).

Considering the putative role of *BRCA1* as a tumour suppressor gene and in keeping with Knudson's two hit model of tumour development (Knudson, 1971), *BRCA1* involvement in the tumorigenic process could also be indirectly demonstrated by allelic loss (Loss of Heterozygosity – LOH) of *BRCA1* linked markers. LOH analysis utilizing such markers facilitates elucidation of the role of *BRCA1* in familial prostate cancer, independent of the type of an inherited predisposing mutation. Indeed, LOH at the *BRCA2* locus is commonly encountered in advanced CaP (Melamed et al, 1997).

To test the notion that *BRCA1* or *BRCA2* are involved in genetic predisposition to CaP, we determined the rate of the predominant germline mutations in 174 unselected Jewish CaP patients and, in addition, searched for allelic losses at the *BRCA1* locus in a subset of patients with a family history of CaP.

*Received 9 July 1999*
*Revised 7 February 2000*
*Accepted 23 March 2000*

*Correspondence to:* E Friedman

### MATERIALS AND METHODS

### Patients' characteristics and tumour material

All patients with a clinical and histopathological diagnosis of CaP who were treated at either the Rabin, Sheba or the Wolfson Medical Centers during 1998, were eligible for participation. The study was approved by the institutional review board (IRB) and all patients signed an informed consent form. All consenting patients filled a detailed questionnaire (available from the authors upon request), that includes demographic data, past medical history, age at diagnosis, family history of cancer, especially breast, ovarian and/or prostate. In addition, for patients who underwent radical retropubic prostatectomy, disease stage and grade were noted. Based on the criteria applied for other familial cancers, at least one first degree relative with prostate or related cancer (breast and ovarian), or more than two second degree relatives with cancer of which one is CaP, breast or ovarian cancer, were classified as familial CaP (Schneider et al, 1983). Paraffin blocks of familial CaP patients were also retrieved.

### DNA extraction

Anticoagulated peripheral blood was withdrawn by venepuncture, and DNA was extracted using standard techniques. DNA was extracted from paraffin embedded tissue as described by Greer and coworkers (Greer et al, 1991), with the slight modification of prolonged incubation at 37°C for 72 hours, with three additions of proteinase K (0.2 mg $ml^{-1}$). Unstained 5–10 μm slides were used for DNA extraction, separating tumorous from non-tumorous tissue from the same slide. The final extraction volume was 150 μl and 3–5 μl were used as template in the polymerase chain reaction (PCR) (see below).

### Mutation analysis of the predominant Jewish mutations in *BRCA1* and *BRCA2*

Mutational analyses for the three predominant mutations (185delAG, 5382InsC in *BRCA1* and 6174delT in *BRCA2*), were carried out by restriction enzyme digest of amplified PCR products using modified amplification primers, to generate novel restriction sites, followed by restriction enzyme analysis to distinguish the mutant from the wild-type allele, as previously described (Abeliovich et al, 1997), and adopted by us (Bar-Sade et al, 1998).

### PCR with chromosome 17 markers for allelic loss determination

PCRs were performed in a final volume of 15 μl containing 3–5 μl template, 10 picomoles of each primer, 200 mM of each TTP, CTP and GTP and 1 mM of ATP, and a radioactively labelled 32P ATP, 10× standard PCR buffer (1.5 mM $MgCl_2$) 0.2 U of thermostable DNA polymerase (Perkin-Elmer Corp., Norwalk, CT). Thermal cycling was accomplished by PTC-100/60 thermocycler (MJ Research Inc., Watertown, MA). The cycling profile included: an initial denaturation at 94°C for 5 minutes, followed by 34 cycles at 55°C for 3 minutes, extension at 72°C for 1 minute and denaturation at 94°C for 45 seconds, with a final extension cycle at 72°C for 5 minutes. Following cycling 4 μl of gel loading buffer (0.025% Bromophemol blue, 0.025% Xylene cyanol and 30% glycerol) were added to the PCR reaction, and 5 μl were loaded onto a 6% sequencing gel, ran for 1.5 to 3 hours at 70W, and autoradiographed for 24 to 72 hours at room temperature using Fuji X-ray films. The microsatellite markers used were previously published with respect to LOH within the *BRCA1* region (Futreal et al, 1994) and were purchased from Research Genetics (Huntsville, AL, USA). Primer loci and designation and their relative linear ordering are as follows: D17S250, D17S579 (both localize centromeric to the *BRCA1* locus), D17S855, D17S1322, D17S1325 (all internal to the *BRCA1* locus) and D17S1323, D17S1327 (both telomeric to the *BRCA1* locus). LOH was scored after visual assessment of the autoradiograms, and the allele sizes were inferred from running an M13 sequence in adjacent lanes.

### Statistical analyses

Comparisons of the rates of the founder Jewish mutations between CaP patients and historical controls (Struewing et al, 1997) were performed using Fisher's exact test.

### RESULTS

### Patients' characteristics

Of 174 consenting patients, 95 (54.6%) were of Ashkenazi origin and the rest ($n$ = 79), non-Ashkenazis. Median age at diagnosis was 66 years (range 45–81 years); In 24/174 (13.8%) diagnosis was made prior to age 55 years, in 19 (10.9%) there was a family history of CaP in a first or second degree relative, and in 12 (6.9%) there was a family history of breast cancer. The majority of patients (105/174, 60%) had stage T2 disease, with 46 (26.4%) with stage 3 disease, and 6 (3.4%) with metastatic disease. About half of the patients (82/174 48.3%) had moderately differentiated disease (Gleason score 5–7), and 12 (6.9%) had poorly differentiated disease (Gleason score 8–10).

### Germline mutational analyses

For technical reasons, mutational analyses were not successful for all mutations in all samples. The presence of the 185delAG *BRCA1* mutation was analysed in 152 patients (87 Ashkenazis), and 2 mutation carriers were detected (1.3% of all patients, 2.3% of the Ashkenazis). The 5382InsC mutation was successfully tested for in 104 patients (60 Ashkenazis) and was detected in 2 patients (1.9% of all patients, 3.3% of the tested Ashkenazis). The 6174delT *BRCA2* mutation was tested in 158 patients (86 Ashkenazis) and one carrier was found (0.6% of all patients, 1.1% of Ashkenazis).

No mutation was detected in any patient in whom the diagnosis was made prior to the age of 55 years, and only one of the carriers (the 6174delT mutation carrier) had a family history of CaP and breast cancer.

### Allelic loss analyses

In 17 of the patients having a family history of CaP or breast cancer, the pathological slides could be retrieved. All 17 patients were informative with at least two polymorphic markers, and at least one of these markers was an intragenic *BRCA1* gene marker. No difference between the allelic pattern of the tumorous and non-tumorous tissue in any of the markers was noted. Figure 1 shows a few examples of the allelic patterns. Notably, non-specific bands

© 2000 Cancer Research Campaign

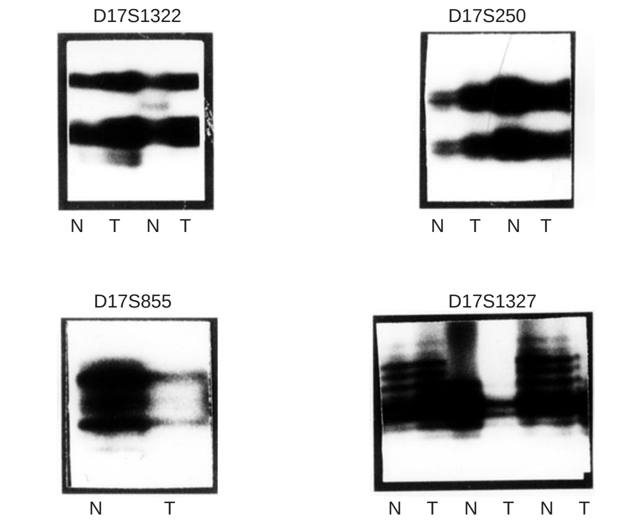

**Figure 1** Representative LOH patterns of four markers from several individuals. The marker tested is shown on the left; the tumorous tissue (T) and the non-tumorous tissue (N) are marked at the bottom. As is evident, allelic patterns and signal intensities are comparable for each marker in the tumour and non-tumorous tissue were present, attributed to the relatively low annealing temperature used. Moreover, the need to use 35 amplification cycles for the paraffin embedded tumour tissue was deemed prohibitive to evaluation of allele intensity as an indicator of allelic loss.

### Statistical analyses

We limited ourselves to analyses of Ashkenazi patients only, since the numbers in the literature refer to this ethnic group. There were no statistically significant differences in the carrier rate of the 185delAG *BRCA1* and 6174delT *BRCA2* mutations between the two groups, and the differences between the general population and the CaP patients was statistically significant for the 5382InsC *BRCA1* mutation (Table 1).

### DISCUSSION

In the present study, the involvement of the *BRCA1* and *BRCA2* genes in inherited predisposition to prostate cancer (CaP) was evaluated by two approaches, direct mutational analyses and allelic loss determination. Jewish Israeli patients with an apparent inherited predisposition to CaP did not display allelic loss involving the *BRCA1* locus on chromosome 17. This conclusion should be drawn tentatively, given the technical limitations of the present study: use of paraffin embedded tissue, no microdissection to separate tumorous from non-tumorous tissue, low annealing temperature, and using 35 PCR cycles. These realities do limit the ability to detect subtle allelic losses, however, the similar allelic patterns displayed in tumour and non-tumorous tissue, might indicate that somatic inactivation of the *BRCA1* gene is infrequent in Jewish familial CaP patients. Given the published rate of 17q allelic loss in sporadic prostate cancer which ranges from 15% (Watanabe et al 1998) to 61% (Deubler et al, 1997), it is surprising that allelic loss was not demonstrated in any of our selected group of patients. It may signify that the underlying molecular mechanisms involved in tumour initiation may follow an alternative pathway in inherited than sporadic CaP. Interestingly, allelic losses

**Table 1** Comparisons between the carrier rate of the predominant Jewish mutations in *BRCA1* and *BRCA2* in the general Jewish Ashkenazi population (Struewing et al, 1997) and the Ashkenazi CaP patients tested in this study

| Mutation (gene) | 185delAG BRCA1 | 5382InsC BRCA1 | 6174delT BRCA2 |
|---|---|---|---|
| CaP | 2/87 (2.3%) | 2/60 (3.3%) | 1/86 (1.1%) |
| General population (1.16%) | 41/5318 (0.77%) | 20/5318 (0.37%) | 59/5087 |
| *P* value | 0.15 (NS) | 0.02 | 0.6 (NS) |

NS, not significant.

at 17q in CaP seem to target a region that lies distal to the *BRCA1* gene, implicating a novel tumour suppressor gene, distinct from the *BRCA1* gene, in CaP tumorigenesis (Williams et al, 1996).

The rate of two of the three predominant Jewish mutations in *BRCA1* and *BRCA2* in Ashkenazi CaP patients did not significantly differ from the rate in the general Jewish Ashkenazi population (Struewing et al, 1997). Furthermore, other parameters usually presumed to be associated with inherited predisposition, such as disease diagnosed prior to age 55 or family history of cancer, were either not present, or affected only one of five germline mutation carriers, respectively. The least common Jewish *BRCA1* mutation, 5382InsC, was statistically more prevalent in CaP patients than in the general population, but this difference is based on a relatively small number of mutation carriers, and should be interpreted cautiously. These data are in agreement with other studies published showing a low rate of involvement of the *BRCA1* and *BRCA2* gene germline mutations in CaP pathogenesis in ethnically diverse populations, and in Ashkenazi Jews, in particular. A study from Washington state reported only one prostate cancer case with a germline *BRCA1* mutation (185delAG) with a questionable family history, and an additional five rare allelic polymorphisms in other familial prostate cancer cases (Langston et al, 1996). An additional study failed to identify an increased risk of breast cancer in relatives of patients with prostate cancer (Issacs et al, 1995). One *BRCA1* germline mutation carrier with CaP and familial cancer was reported among 24 Japanese CaP patients, no 185delAG or 6174delT mutation carriers were detected among 60 Ashkenazi CaP patients (Lehrer et al, 1998), and one 6174delT *BRCA2* mutation carrier was found among 47 Jewish Ashkenazi individuals from 18 families with familial CaP (Wilkens et al, 1999). Recently, three mutation carriers (two with the 185delAG and one harbouring 6174delT) were identified among 87 unselected prostate cancer patients from Israel (Hubert et al, 1999). Interestingly, three of 87 age-matched healthy controls were also mutation carriers. These direct mutational analyses studies are not in line with the reported increased relative risk for developing CaP among patients with a family history of breast and ovarian cancer. Furthermore, in an Icelandic population, where a single predominant mutation (999del5) in *BRCA2* exists, the mutation was six times more prevalent among CaP patients (2.7%) as among the general population (0.4%) (Johannesdottir et al, 1996). In addition, if we limit our analysis to the Ashkenazi patients studied herein, the overall carrier rate of one of the predominant mutations is 5/87 (5.7%), a rate double that of the general Ashkenazi population.

In conclusion, in Jewish CaP patients, germline mutations in the *BRCA1* and *BRCA2* genes seem to contribute little to the tumorigenic process, and somatic inactivation of the *BRCA1* genes

© 2000 Cancer Research Campaign occurs infrequently in familial Jewish CaP patients. From the current available data it seems that men harbouring one of the predominant Jewish germline mutations do not have an increased risk for developing early onset CaP. The lifetime risk for CaP development in Jewish *BRCA1* and *BRCA2* mutation carriers and the occurrence rate of these mutations in unselected CaP patients remains to be determined in a larger, prospective study.

### REFERENCES

Abeliovich D, Kaduri L, Lerer I et al (1997) The founder mutation 185delAG and 5382insC in BRCA1 and 617delT in BRCA2 appear in 60% of ovarian cancer and 30% of early-onset breast cancer among Ashkenazi women. *Am J Hum Genet* **60**: 505–514

Anderson DE and Badzioch MD (1993) Familial effects of prostate and other cancers on lifetime breast cancer risk. *Breast Cancer Res Treat* **28**: 107–113

Arason A, Barkadottier RB and Egilsson V (1993) Linkage analysis of chromosome 17 markers and breast ovarian cancer in Icelandic families, and possible relationship to prostatic cancer. *Am J Hum Genet* **52**: 711–717

Bar-Sade RB, Kruglikova A, Modan B et al (1998) The 185delAG BRCA1 mutation originated before the dispersion of Jews in the diaspora and is not limited to Ashkenazim. *Hum Mol Genet* **7**: 801–805

Briana JW, Emma J, Xiao LZ et al (1996) Evidence for a tumor suppressor gene distal to BRCA1 in prostate cancer. *J Urol* **155**: 720–725

Carter BS, Beatty TH, Steinberg GD, Childs B and Walsh PC (1992) Mendelian inheritance of familial prostate cancer. *Proc Natl Acad Sci USA* **89**: 3367–3371

Deubler DA, Williams BJ, Zhu XL, Steele MR, Rohr LR, Jensen JC, Stephenson RA, Changus JE, Miller GJ, Becich MJ and Brothman AR (1997) Allelic loss detected on chromosomes 8, 10 and 17 by fluorescence in situ hybridization using single-copy P1 probes on isolated nuclei from paraffin-embedded prostate tumors. *Am J Pathol* **150**: 841–850

Easton DF, Ford D, Bishop DT and the Breast Cancer Linkage Consortium (1995) Risks of cancer in BRCA1 mutation carriers. *Am J Hum Genet* **56**: 265–271

Easton DF, Steele L, Fields P et al (1997) Cancer risks in two large breast cancer families linked to BRCA1 on chromosome 13q12–13. *Am J Genet* **61**: 120–128

Ford D, Easton DF, Bishop DT, Narod SA and Goldgar DE (1994) The breast cancer linkage consortium. Risks of cancer in BRCA1 mutation carriers. *Lancet* **343**: 692–695

Futreal PA, Cochran C, Rosenthal J, et al (1994) Isolation of a diverged homeobox gene, MOX1, from the BRCA1 region on 17q21 by solution hybrid capture. *Hum Mol Genet* **3**: 1359–1364

Gittes RF (1991) Carcinoma of the prostate. *N Engl J Med* **324**: 236–245

Greer CE, Peterson SL, Kiviat NB and Manos MM (1991) PCR amplification from paraffin embedded tissues. *Am J Clin Pathol* **95**: 117–124

Hubert A, Peretz T, Manor O, Kaduri L, Wienberg N, Lerer I, Sagi M and Abeliovich D (1999) The Jewish Ashkenazi founder mutations in the BRCA1/BRCA2 genes are not found at an increased frequency in Ashkenazi patients with prostate cancer. *Am J Hum Genet* **65**: 921–924

Issacs SD, Kiemeney LALM, Baffoe-Bonnie A, Beaty TH and Walsh PC (1995) Risk of cancer in relatives of prostate cancer probands. *J Natl Cancer Inst* **87**: 991–996

Johannesdottir G, Gudmundsson J, Bergthorsson JT, Arason A, Agnarsson BA, Eiriksdottir G, Johannsson OT, Borg A, Ingvarsson S, Easton DF, Egilsson V and Barkardottir RB (1996) High prevalence of the 999del5 mutation in Icelandic breast and ovarian cancer patients. *Cancer Res* **56**: 3663–3665

Knudson AG (1971) Mutation and cancer: Statistical study of retinoblastoma. *Proc Natl Acad Sci USA* **68**: 820–823

Langston AA, Stabford JL, Wicklund KG, Thompson JD, Blazej RG and Ostrander EA (1996) Germ-line BRCA1 mutations in selected men with prostate cancer. *Am J Hum Genet* **58**: 881–885

Lehrer S, Fodor F, Stock RG, Stone NN, Eng C, Song HK and McGovern M (1998) Absence of 185delAG mutation of the BRCA1 gene and 6174delT mutation of the BRCA2 gene in Ashkenazi Jewish men with prostate cancer. *Br J Cancer* **78**: 771–773

Melamed J, Einhorn JM and Ittmann MM (1997) Allelic loss on chromosome 13q in human prostate carcinoma. *Clin Cancer Res* **3**: 1867–1872

Schneider NR, Chaganti SR, German J and Chagnti RSK (1983) Familial predisposition to cancer and age at onset of disease in randomly selected cancer patients. *Am J Hum Genet* **35**: 454–467

Sellers TA, Potter JD, Rich SS, Drinkard CR, Bostick RM, Kushi LH, Zheng W and Folsom AR (1994) Familial clustering of breast and prostate cancers and risk for postmenopausal breast cancer. *J Natl Cancer Inst* **86**: 1860–1865

Sheldon DN, Williams M and Fraley EE (1980) Incidental carcinoma of the prostate: a review of the literature and critical reappraisal of classification. *J Urol* **124**: 626–631

Sigurdsson K, Thorlacius S, Tomasson J et al (1997) BRCA1 mutation in Icelandic prostate cancer patients. *J Mol Med* **75**: 758–761

Struewing JP, Hartge P, Wacholder S et al (1997) The risk of cancer associated with specific mutations of BRCA1 and BRCA2 among Ashkenazi Jews. *N Engl J Med* **336**: 1401–1408

Tulinius H, Egilsson V, Olafsdottir GH and Sigvaldason H (1992) Risk of prostate, ovarian and endometrial cancer among relatives of women with breast cancer. *Br Med J* **305**: 855–857

Watanabe M, Shiraishi T, Muneyuki T, Nagai M, Fukutome K, Murata M, Kawamura J and Yatani R (1998) Allelic loss and microsatellite instability in prostate cancers in Japan. *Oncology* **55**: 569–574

Whittemore AS, Wu AH, Kolonel AN, John EM, Gallagher RP, Howe GR, West DW, The CZ and Stamey T (1995) Family history and prostate cancer risk in black, white, and Asian men in the United States and Canada. *Am J Epidemiol* **141**: 732–740

Wilkens EP, Freije D, Xu J, Nusskern DR, Suzuki H, Isaacs SD, Wiley K, Bujnovsky P, Meyers DA, Walsh PC and Isaacs WB (1999) No evidence for a role of BRCA1 or BRCA2 mutations in Ashkenazi Jewish families with hereditary prostate cancer. *Prostate* **39**: 280–284

Williams BJ, Jones E, Zhu XL, Steele MR, Stephenson RA, Rohr LR and Brothman AR (1996) Evidence for a tumor suppressor gene distal to BRCA1 in prostate cancer. *J Urol* **155**: 720–725

 *© 2000 Cancer Research Campaign*
